# Electron-triggered chemistry in HNO_3_/H_2_O complexes†
†Electronic supplementary information (ESI) available: Mass spectrum from electron ionization of H_2_O/HNO_3_ complexes, benchmark calculations, and the calculated cluster structures. See DOI: 10.1039/c7cp01205e



**DOI:** 10.1039/c7cp01205e

**Published:** 2017-03-24

**Authors:** Jozef Lengyel, Milan Ončák, Juraj Fedor, Jaroslav Kočišek, Andriy Pysanenko, Martin K. Beyer, Michal Fárník

**Affiliations:** a J. Heyrovský Institute of Physical Chemistry v.v.i. , Czech Academy of Sciences , Dolejškova 3 , 18223 Prague , Czech Republic . Email: michal.farnik@jh-inst.cas.cz; b Institut für Ionenphysik und Angewandte Physik , Leopold-Franzens-Universität Innsbruck , Technikerstraße 25 , 6020 Innsbruck , Austria . Email: jozef.lengyel@uibk.ac.at

## Abstract

Polar stratospheric clouds, which consist mainly of nitric acid containing ice particles, play a pivotal role in stratospheric chemistry. We investigate mixed nitric acid–water clusters (HNO_3_)_*m*_(H_2_O)_*n*_, *m* ≈ 1–6, *n* ≈ 1–15, in a laboratory molecular beam experiment using electron attachment and mass spectrometry and interpret our experiments using DFT calculations. The reactions are triggered by the attachment of free electrons (0–14 eV) which leads to subsequent intracluster ion–molecule reactions. In these reactions, the nitrate anion NO_3_^–^ turns out to play the central role. This contradicts the electron attachment to the gas-phase HNO_3_ molecule, which leads almost exclusively to NO_2_^–^. The nitrate containing clusters are formed through at least three different reaction pathways and represent terminal product ions in the reaction cascade initiated by the electron attachment. Besides, the complex reaction pathways represent a new hitherto unrecognized source of atmospherically important OH and HONO molecules.

## Abstract 2

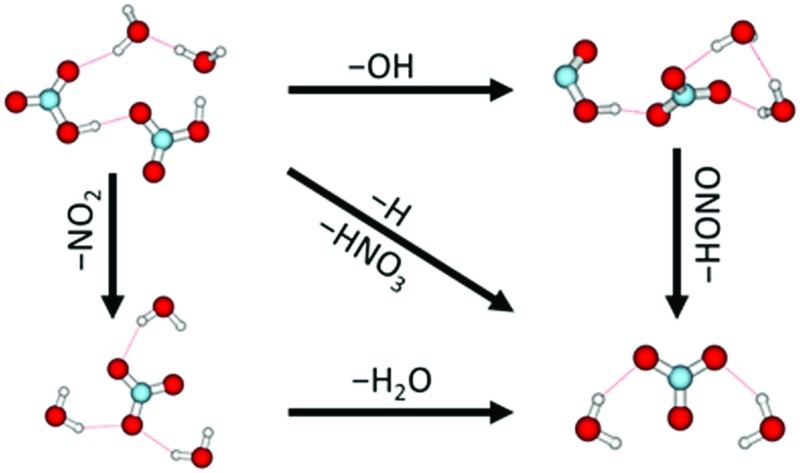
Electron attachment to mixed HNO_3_/H_2_O clusters yields several atmospherically relevant species such as NO_3_^–^, HONO and OH radical.

## Introduction

Heterogeneous chemistry involving ice particles, cloud droplets and aerosols plays an important, albeit not yet fully understood, role in the atmospheric chemistry.^1^ For example, polar stratospheric clouds (PSCs) provide an environment for heterogeneous reactions of chlorine activation on their surface, which lead to more than 90% of the ozone depletion.^2^ From the chemical perspective, PSCs mostly consist of nitric acid/ice particles.^2–4^ Under the specific conditions of the upper atmosphere, chemical reactions are often initiated either by photons or electrons. Free electrons originate from, *e.g.*, photoionization, solar and magnetospheric particles or cosmic rays and the corresponding secondary ionization processes. Typically, the electrons are effectively thermalized to low energies (<1 eV) *via* multiple inelastic collisions.^5^ At these low energies, they can attach to molecules and clusters in the atmosphere.

The gas-phase reactions between free electrons and HNO_3_ molecules have been studied only using flowing afterglow techniques,^6–8^ and we are not aware of any crossed-beam electron attachment data. It has been initially concluded^6^ that the dissociative electron attachment (DEA) in HNO_3_ leads only to the NO_2_^–^ product ion which is the most exothermic channel with a reaction enthalpy of Δ_r_ *H*_298K_ = –0.13 eV. Recently, Shuman *et al.* ^8^ reported a weak endothermic channel producing OH^–^ (Δ_r_ *H*_298K_ = 0.31 eV). The formation of NO_3_^–^ is rather endothermic (Δ_r_ *H*_298K_ = 0.45 eV) and has not been observed experimentally so far. In complexes with ice nanoparticles, the outcome of the electron-induced reactions can be significantly affected by the environment and by acidic dissociation of HNO_3_.

In the present experiment, we produce mixed (HNO_3_)_*m*_(H_2_O)_*n*_ clusters by supersonic expansions of HNO_3_/H_2_O vapor mixtures at different temperatures and buffer gas pressures. We analyzed the composition of the clusters generated under these conditions in our previous investigations with electron-impact ionization and sodium doping.^9,10^ These clusters then interact with the electron beam and the negative ion production is monitored by a mass spectrometer. In order to provide a reference gas-phase spectrum, we have also performed electron beam attachment experiments to gas-phase HNO_3_. Employing quantum chemical methods, we characterize the small neutral complexes of (HNO_3_)_*m*_(H_2_O)_*n*_, calculate the energetics of the initial dissociation step, *i.e.* the generation of NO_2_^–^, NO_3_^–^ or OH^–^ from HNO_3_, and sample the potential energy surface to locate the energetically most stable isomers and intracluster rearrangements.

## Experimental and theoretical methods

### Experimental part

The experiments were carried out on the CLUster Beam (CLUB) apparatus in Prague.^11–13^ The mixed HNO_3_/H_2_O clusters were generated in a home-build source *via* continuous supersonic expansion of the HNO_3_ solution (70%, Sigma-Aldrich) and helium (99.996%, Messer) as a buffer gas through a divergent conical nozzle of 100 μm diameter, 2 mm long, and approximately 30° full opening angle into the vacuum. The clustering conditions were controlled by heating the HNO_3_ solution in the reservoir and by the stagnation pressure of the buffer gas. Reservoir temperatures between 60 and 80 °C and buffer gas pressures between 1 and 2 bar were employed. The buffer gas carried the HNO_3_/H_2_O vapor to the nozzle heated independently to a higher temperature (usually by 5–10 °C) to avoid any condensation. However, changing the source conditions within the present range had little effect on the mass spectra. We have analyzed the composition of the neutral clusters previously from the positive mass spectra.^9,10^ From this analysis, the present neutral clusters have a mixed composition of (HNO_3_)_*m*_(H_2_O)_*n*_, with *m* ≈ 1–6 and *n* ≈ 1–15.

The cluster beam was skimmed and passed through three differentially pumped chambers before entering the ion source of the perpendicularly mounted reflectron time-of-flight mass spectrometer. The voltages on the extraction plates were set to detect negative ions. The (HNO_3_)_*m*_(H_2_O)_*n*_ clusters were ionized using free electrons (0–14 eV) from a pulsed electron gun at 10 kHz frequency during 2 μs in the extraction region of the spectrometer. After 0.5 μs delay to exclude the effects of any free electrons, a 2 μs extraction pulse was applied to extract the negative ions, which were detected on the Photonics MCP detector in the Chevron configuration. The electron attachment experiment was described in our recent paper,^12^ typical electron current at the present experiment was 0.2 μA at 5 eV. The electron-energy scale was calibrated using the 2.2 eV resonance in the O^–^ production from N_2_O. The individual mass spectra recorded with an electron energy step of 0.25 eV in the range 0–14 eV allow for the evaluation of the electron energy dependence for each individual peak in the mass spectra, however, a detailed analysis of the electron attachment efficiency dependence is beyond the scope of the present study and will be the subject of future work.

The HNO_3_ gas-phase data were recorded on another apparatus: dissociative electron attachment spectrometer.^14^ The electron beam is produced in a trochoidal electron monochromator and passes through a collision chamber filled with the studied gas. The electron current is monitored by a Faraday cup located behind the collision cell. The entire experiment is pulsed: the electrons pass the target chamber during 200 ns while it is field-free and after additional 200 ns (when the electrons leave the chamber) a negative voltage of –300 V is pulsed across the chamber which pushes the anions formed in the cell towards the ion time-of-flight mass analyzer in the direction perpendicular to the electron beam. The anions are detected by a microchannel plate, counted, and their arrival times are analyzed.

**Fig. 1:**
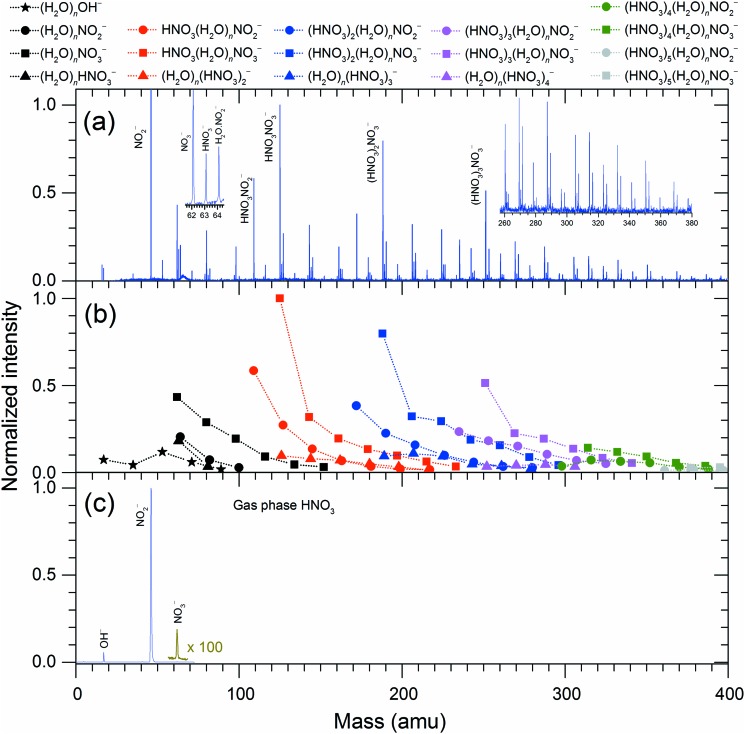
Panels (a and c): cumulative negative ion mass spectra for (HNO_3_)_*m*_(H_2_O)_*n*_ cluster beam and gas-phase HNO_3_, respectively. Panel (b): cluster beam spectral analysis distinguishing different groups of mass peaks. The cluster beam spectrum was obtained as a sum of all the mass spectra in the energy range 0–14 eV with an energy step of 0.25 eV. It was normalized to the most intense cluster ion HNO_3_NO_3_^–^ (*m/z* = 125), the NO_2_^–^ peak (intensity of 3.5) is omitted from the figure for clarity. The gas-phase spectrum represents a sum of the mass spectra in the same energy range with a step of 0.05 eV, normalized to the NO_2_^–^ peak.

The target chamber is filled with the vapor of concentrated nitric acid solution in water (70%). The vapor thus consists of a mixture of gas-phase HNO_3_ and H_2_O molecules. The presence of water vapor in the sample does not interfere with the results shown in Fig. 1: the DEA to H_2_O leads to H^–^ and O^–^ production,^15^ none of the fragments reported form HNO_3_ (NO_2_^–^, OH^–^, NO_3_^–^) originate from water. The spectra recorded after a longer time (tens of minutes) after the pump–freeze–thaw purification of the sample contained a weak O^–^ signal. The electron-energy dependence of this signal was identical to that of O^–^ originating from DEA to NO_2_.^16^ Since HNO_3_ is known to release NO_2_, we conclude that the detected O^–^ does not originate from DEA to HNO_3_.

### Computational details

Interpretation of the experimental results was supported by DFT calculations. We included the following clusters: (HNO_3_)_*m*_(H_2_O)_*n*_, *m* = 1–3, *n* = 0–5, (HNO_3_)_*m*_(H_2_O)_*n*_NO_2_^–^, *m* = 0–2, *n* = 0–5, (HNO_3_)_*m*_(H_2_O)_*n*_NO_3_^–^, *m* = 0–2, *n* = 0–5 (also *n* = 6 for *m* = 0, 1), (H_2_O)_*n*_OH^–^, *n* = 0–5. For all clusters with more than two composing molecules, molecular dynamics was run to find the most stable isomers, using the BLYP/6-31+g* method, with a time step of 40 a.u. (∼1 fs). First, 10 000 steps were performed at 400 K, then another 10 000 steps at 200 K starting from the last geometry of the first molecular dynamics run. The temperature was maintained by a Nosé–Hoover chain of 4 thermostats with a relaxation time of 0.0015 a.u. The structures were re-optimized at the M06-2X/aug-cc-pVDZ level after every 1000 steps; the M06-2X functional was chosen due to its performance for flexible anionic clusters.^17^ Only the most stable isomers were considered, the respective structures are shown in Fig. S2 (ESI†). For HNO_3_(H_2_O)_*n*_, *n* = 4, 5, ion pair structures were also included, based on the structures presented elsewhere.^18^ Localized structures are in good agreement with those found by other studies.^18–21^ All reported energies were calculated at the M06-2X/aug-cc-pVDZ level and include zero-point-energy corrections (all structures represent local minima). Electronic structure calculations were performed using the Gaussian09 program,^22^ the ABIN code was used for molecular dynamics.^23^ Further details and benchmark calculations can be found in the ESI.†

## Results and discussion

Fig. 1 summarizes the experimental measurements of the DEA to HNO_3_/H_2_O complexes and gas-phase HNO_3_. The gas-phase spectrum of HNO_3_ (Fig. 1(c)) is dominated by a NO_2_^–^ fragment (as outlined above, the NO_2_^–^ + OH dissociation channel is the only exothermic one), a weak OH^–^ fragment and, for the first time detected, a NO_3_^–^ fragment. The NO_2_^–^ : OH^–^ : NO_3_^–^ intensity ratio is 96.5 : 3.4 : 0.03. The ratio of the first two fragments is in very good agreement with that observed in mass spectrometry of flowing afterglow plasma,^8^ the electron energies in that type of experiment, however, were probably too low to reach the threshold for NO_3_^–^ production.

**Fig. 2:**
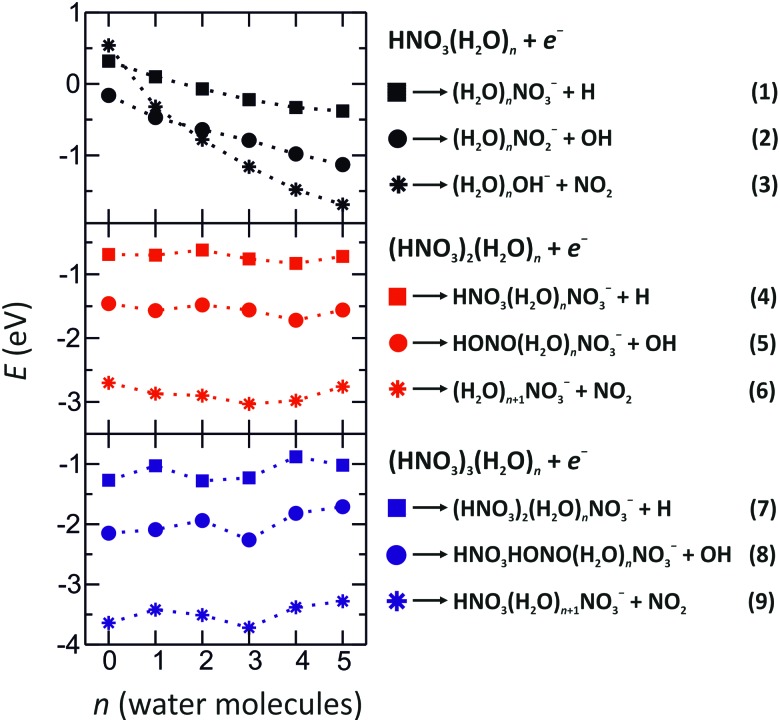
Reaction energies of various dissociation channels after electron attachment to (HNO_3_)_*m*_(H_2_O)_*n*_ clusters, *m* = 1–3, *n* = 0–5, calculated at the M06-2X/aug-cc-pVDZ level of theory.

The cluster beam spectrum (Fig. 1(a and b)) is also dominated by an intense peak of NO_2_^–^. This is not surprising as a part of the present signal originates from HNO_3_ monomers in the beam. However, among the complex anions (with the mass higher than the HNO_3_ monomer), the dominant species are ions containing NO_3_^–^. The total intensity ratio of peaks from complexes containing NO_3_^–^ : NO_2_^–^ : HNO_3_^–^ : OH^–^ is 57 : 32 : 8 : 2. The dramatic increase in the NO_3_^–^ anion signal with respect to the spectrum of gas-phase HNO_3_ indicates rich intracluster chemistry. In the gas-phase reactions, the NO_3_^–^ anion is often seen as the terminal product of ion–molecule reactions involving HNO_3_.^6,24–28^ In order to elucidate the pathways leading to its production in the present species, Fig. 2 shows energies for various intracluster reactions following several initial dissociation steps and summarizes the suggested pathways (1)–(9).

It should be noted that the efficiency of DEA channels does not primarily depend on asymptotic energetics.^29,30^ Electron attachment is a vertical process and the decisive factor for breaking specific bonds, in case of a prompt dissociation, is often the direction of the gradient of the anionic potential energy surface at the initial structure. However, in systems with sufficient degrees of freedom such as clusters, the excitation energy may be redistributed over the vibrational degrees of freedom, with the decay proceeding statistically. In this case, the product distribution is dominated by the energetics of the dissociation channels. We identify three mechanisms leading to NO_3_^–^ production, one proceeding in the first described way, *i.e.* kinematically driven, the other two driven by the asymptotic energetics.

### Pathways leading to NO_3_^–^-containing complexes

#### NO_3_^–^ production *via* DEA to acidically dissociated clusters

In the neutral state, before any interaction with free electrons, HNO_3_ can acidically dissociate in sufficiently large water clusters to form a nitrate anion NO_3_^–^ and a hydronium cation H_3_O^+^:

10a (H_2_O)_*n*_(HNO_3_)_*m*_ → H_3_O^+^(H_2_O)_*n*–1_(HNO_3_)_*m*–1_NO_3_^–^

Previous experiments^9,18,31–33^ showed that the acidic dissociation (10a) might occur in (H_2_O)_*n*_HNO_3_ clusters with *n* ≥ 4–5. At the M062X/aug-cc-pVDZ level, the acidically dissociated clusters are less stable by 0.01 eV for *n* = 4 and more stable by 0.11 eV for *n* = 5 with respect to the non-dissociated ones. For (H_2_O)_*n*_(HNO_3_)_*m*_ species, the formation of the NO_3_^–^···H_3_O^+^ ion pair is predicted to be energetically preferred for 3 and 4 water molecules for *m* = 2 and 3, respectively, see Fig. S2 in the ESI.† This is also consistent with the interpretation of the previously measured mass spectra of positively charged ions.^9,31^ Previous *ab initio* molecular dynamics showed that acidic dissociation immediately leads to the formation of a “solvent-separated” ion pair and the proton migrates across the cluster even on cold ice particles.^34^

Collision of the neutral cluster containing an H_3_O^+^/NO_3_^–^ ion pair with a free electron will most probably lead to recombination, forming a metastable H_3_O radical that subsequently dissociates, yielding a hydrogen atom and water.^35^ The energy released upon recombination will lead to the evaporation of the hydrogen atom, which is only weakly bound:

10b H_3_O^+^(H_2_O)_*n*_(HNO_3_)_*m*–1_NO_3_^–^ + e^–^ → (H_2_O)_*n*+1_(HNO_3_)_*m*–1_NO_3_^–^ + H

The acidic dissociation increases both the dipole moment and the polarizability of the neutral clusters.^36^ The cross section for electron capture rapidly increases with the strength of the long-range electron–target interaction,^37^ one can thus assume a high probability of reaction (10b).

#### NO_2_^–^ production, followed by cluster rearrangement

The NO_2_^–^ production from gas-phase HNO_3_ *via* DEA is the most prominent channel due to its exothermicity. Upon hydration of HNO_3_, the exothermicity of this channel (top panel of Fig. 2) increases due to the interaction of NO_2_^–^ with water. In solvated clusters with more than one HNO_3_ molecule, NO_2_^–^ can react with another HNO_3_ molecule forming HONO and NO_3_^–^, reactions (5) and (8).

Since the mass of (HNO_3_)_*m*_(H_2_O)_*n*_NO_2_^–^ is equal to that of HONO(HNO_3_)_*m*–1_(H_2_O)_*n*_NO_3_^–^, these ions cannot be distinguished in the mass spectrometry experiment. However, our molecular dynamics calculations indicate that the HONO formation proceeds irreversibly. The gas-phase reaction HNO_3_ + NO_2_^–^ → NO_3_^–^ + HONO has an energy of –0.74 eV (calculated at the M06-2X/aug-cc-pVDZ level of theory). The considerably larger acid strength of HNO_3_ compared to that of HONO suggests that the proton transfer reaction from HNO_3_ to NO_2_^–^ can be expected in small mixed clusters (see Table S3 in the ESI†). The final cluster anion composition will also depend on whether HONO stays adsorbed on the cluster or evaporates:

11 HONO(HNO_3_)_*m*–2_(H_2_O)_*n*_NO_3_^–^ → (HNO_3_)_*m*–2_(H_2_O)_*n*_NO_3_^–^ + HONO

The calculated evaporation energies of H_2_O, HONO and HNO_3_ from the anionic complexes are compared in Table 1. It can be seen that the evaporation of a HONO molecule is in all cases more energetically demanding than water evaporation. However, the evaporation energies of HONO do not differ significantly from that of H_2_O for several cluster sizes. Energetically the most demanding and therefore the least probable is the evaporation of HNO_3_.

**Table 1:** Energy of evaporation (in eV) of H_2_O, HONO, and HNO_3_ from clusters formed after OH dissociation and internal rearrangement to form HONO. Calculated at the M06-2X/aug-cc-pVDZ level of theory

Ion	Evaporating molecule	*n*					
		0	1	2	3	4	5
HONO(H_2_O)_*n*_NO_3_^–^	H_2_O	—	0.48	0.37	0.41	0.64	0.45
	HONO	1.00	0.85	0.68	0.60	0.78	0.79
HONO(H_2_O)_*n*_HNO_3_NO_3_^–^	H_2_O	—	0.54	0.23	0.67	0.40	0.46
	HONO	0.50	0.66	0.51	0.70	0.54	0.51
	HNO_3_	0.95	1.00	0.86	1.12	0.88	0.89

#### OH^–^ production, followed by cluster rearrangement

Upon hydration of one HNO_3_ molecule (top panel of Fig. 2), the formation of (H_2_O)_*n*_OH^–^ becomes the most exothermic channel for *n* > 1. As shown previously, it is more exothermic than the (H_2_O)_*n*_NO_2_^–^ channel due to the stronger interaction of water with OH^–^ than with the NO_2_^–^ ion.^38^ If more than one HNO_3_ molecule is present in the neutral complex, the OH^–^ anion reacts rapidly with HNO_3_ and forms NO_3_^–^, reactions (6) and (9).

Again, such intracluster rearrangement cannot be followed by the mass spectrometry experiment. In our MD simulations, this reaction is seen to proceed spontaneously within 1 ps. It is strongly preferred already in the gas phase, the calculated energy of the corresponding HNO_3_ + OH^–^ → NO_3_^–^ + H_2_O reaction is –3.05 eV. With an increasing degree of hydration, the reaction retains its exothermic character, see ref. 27 and Table S3 in the ESI.†

We cannot determine the relative importance of these three channels leading to NO_3_^–^ containing complexes as there are factors favoring each of them. As described above, a significant portion of neutral complexes will be acidically dissociated, leading to the first proposed channel. However, it is reasonable to assume that these will yield exclusively NO_3_^–^-containing anions, since NO_3_^–^ is already present in the cluster before the interaction with the free electron takes place, and the electron is attracted by the positive charge center H_3_O^+^. The significant abundance of complexes containing NO_2_^–^ and HNO_3_^–^ in Fig. 1 suggests that the dissociation *via* the first channel is not the only process occurring. The fact favoring the second channel is that its first step, DEA to HNO_3_ yielding NO_2_^–^, has a high cross section. The third channel is the most exothermic one, it however requires OH^–^ production as its first step, which has a low probability for the gas-phase HNO_3_ (Fig. 1(c)).

### Pathways leading to NO_2_^–^, HNO_3_^–^ and OH^–^-containing complexes

The second strongest progression in Fig. 1 are from the clusters containing NO_2_^–^. The formation of (H_2_O)_*n*_NO_2_^–^ clusters is predicted to be the most exothermic channel for *n* < 2. With increasing degree of hydration, the formation of (H_2_O)_*n*_OH^–^ prevails energetically as discussed above. For complexes with more than one HNO_3_ molecule, reactions (5) and (8) have an energy of about –1.5 eV and –2.0 eV for 2 and 3 HNO_3_ molecules, respectively. The formation of NO_2_^–^ is then followed by an intracluster rearrangement to form HONO and NO_3_^–^.

Anions of the nominal composition (H_2_O)_*n*_(HNO_3_)_*m*_^–^ represent 8% of the total signal. For small cluster sizes (*n* ≤ 4), HNO_3_ is not ionically dissociated in the neutral cluster. Moreover, HNO_3_ has an appreciable electron affinity of 0.56 ± 0.17 eV.^39^ HNO_3_^–^ valence anions are stable, decomposition into NO_2_^–^ + OH is mildly exothermic. It is therefore entirely plausible that (H_2_O)_*n*_(HNO_3_)_*m*_^–^ clusters contain a valence-bound HNO_3_^–^ anion, which may decompose upon hydration with a small number of water molecules. In the hydrogen bonded network, it is also possible that NO_2_^–^ + OH dissociation products remain in the cluster if the dissociation is not exothermic enough to cause product evaporation.

The (H_2_O)_*n*_OH^–^ cluster series represents about 2% of the total signal. Its presence can be explained by (H_2_O)_*n*_(HNO_3_)_*m*_^–^ dissociation into OH^–^ + NO_2_, with NO_2_ not forming strong hydrogen bonds. This would be in line with our recent work,^38^ where OH^–^ formation is observed in binary collisions of hydrated electrons with HNO_3_ in the gas phase. Electron attachment to a neutral (H_2_O)_*n*_HNO_3_ cluster would form the same initial state, a hydrated valence-bound HNO_3_, which quickly undergoes O–N bond cleavage to form (H_2_O)_*n*_OH^–^.

### Atmospheric relevance

**Fig. 3:**
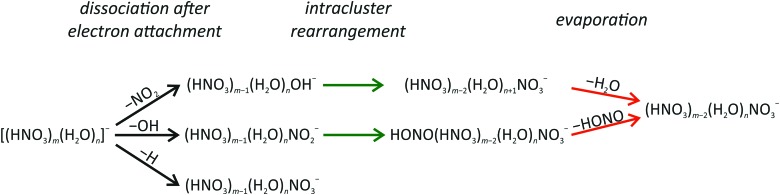
Schematic illustration of reaction pathways observed in electron attachment to mixed (HNO_3_)_*m*_(H_2_O)_*n*_ clusters.

The present experiment shows that low energy free electrons are efficiently trapped in the mixed HNO_3_/H_2_O clusters. The electrons immediately initiate reactions with HNO_3_ through different reaction channels. Fig. 3 summarizes the processes occurring after DEA to mixed HNO_3_/H_2_O clusters. For clusters with one HNO_3_ molecule, three different fragment series (with OH^–^, NO_2_^–^ or NO_3_^–^ anions) can be produced. For clusters with more than one HNO_3_ molecule, clusters containing a NO_3_^–^ core anion play the central role and represent the terminal product for all considered dissociation channels.

There is a close analogy between the composition of the studied clusters and PSCs. Therefore, our results can have important implications for the impact of HNO_3_ on heterogeneous chemistry. In particular, we suggest that DEA to molecules in PSC particles results in the formation of NO_3_^–^ ions, which are among the most abundant anions in the stratosphere. As an example, (HNO_3_)_2_NO_3_^–^ dominates among all negative stratospheric ions in the PSC region at heights below 30 km.^40–42^ We show that although DEA to HNO_3_/H_2_O particles leads to a cascade of reactions yielding reactive OH and HONO molecules, it is terminated by relatively stable NO_3_^–^. It is unlikely that this ion takes part in further reactions, and it rather promotes nucleation of new particles.^43^ However, the OH and HONO molecules released from the mixed nitric acid–water clusters upon the electron attachment play a pivotal role in the atmospheric chemistry. The atmospheric models do not account for the total yield of HONO in the atmosphere.^44^ It was indicated that a further, hitherto unrecognized, source of HONO must exist in the atmosphere and heterogeneous chemistry involving atmospheric aerosol particles was suspected to represent such a source. The electron attachment to the nitric acid–water clusters and our suggested reaction pathways represent one of the possible HONO sources.

## Conclusions

In order to mimic electron-triggered reactions in PSC particles we investigated electron attachment to mixed nitric acid–water clusters (HNO_3_)_*m*_(H_2_O)_*n*_, *m* = 1–6, *n* = 1–15, in a crossed beam experiment. The products were analyzed using negative ion mass spectrometry and the results were interpreted by DFT calculations. The reactions induced by the electron attachment in the range of 0–14 eV yielded predominantly NO_3_^–^ ion containing species through different reaction pathways. This is in contrast to both the electron attachment to gas-phase HNO_3_, yielding primarily NO_2_^–^, and the reaction of gas-phase HNO_3_ with a hydrated electron, which leads to the formation of hydrated OH^–^ clusters. The initial driving force in the present DEA to HNO_3_/H_2_O complexes is the proton transfer to form the H_3_O^+^/NO_3_^–^ ion pair where the electron recombines with the proton, yielding the hydrogen atom and NO_3_^–^. The NO_3_^–^ anion represents the terminal product ion also in the intracluster reaction pathways driven by the energetics, which yield also OH and HONO molecules.
