# Supplementary material for: Electron-triggered chemistry in HNO3/H2O complexes
Source: Phys Chem Chem Phys. 2017 Mar 24;19(19):11753–8. doi: 10.1039/c7cp01205e (PMC5450009; doi:10.1039/c7cp01205e)
Supplement: Supplementary file 1 [file CP-019-C7CP01205E-s001.pdf]

Electronic Supplementary Material (ESI) for Physical Chemistry Chemical Physics.
This journal is © the Owner Societies 2017

Electronic Supplementary Information for article:

Electron-triggered chemistry in HNO_3/H_2O complexes

Jozef Lengyel,*[ab] Milan Ončák,[b] Juraj Fedor,[a] Jaroslav Kočišek,[a] Andriy Pysanenko,[a] Martin K. Beyer[b] and Michal Fárník*[a]

[a] *J. Heyrovský Institute of Physical Chemistry v.v.i., Czech Academy of Sciences, Dolejškova 3, 18223 Prague, Czech Republic.*

[b] *Institut für Ionenphysik und Angewandte Physik, Leopold-Franzens-Universität Innsbruck, Technikerstraße 25, 6020 Innsbruck, Austria.*

Corresponding author(s): jozef.lengyel@uibk.ac.at, michal.farnik@jh-inst.cas.cz

Mass spectrum

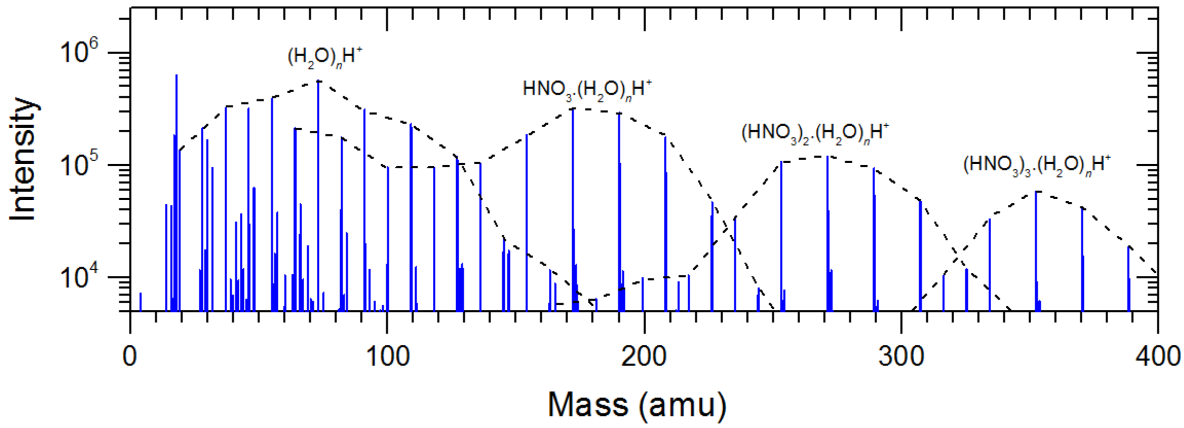

Figure S1. Positive ion mass spectrum of the $(HNO_3)_m(H_2O)_n$ clusters at 70 eV electron energies.

Computational Details

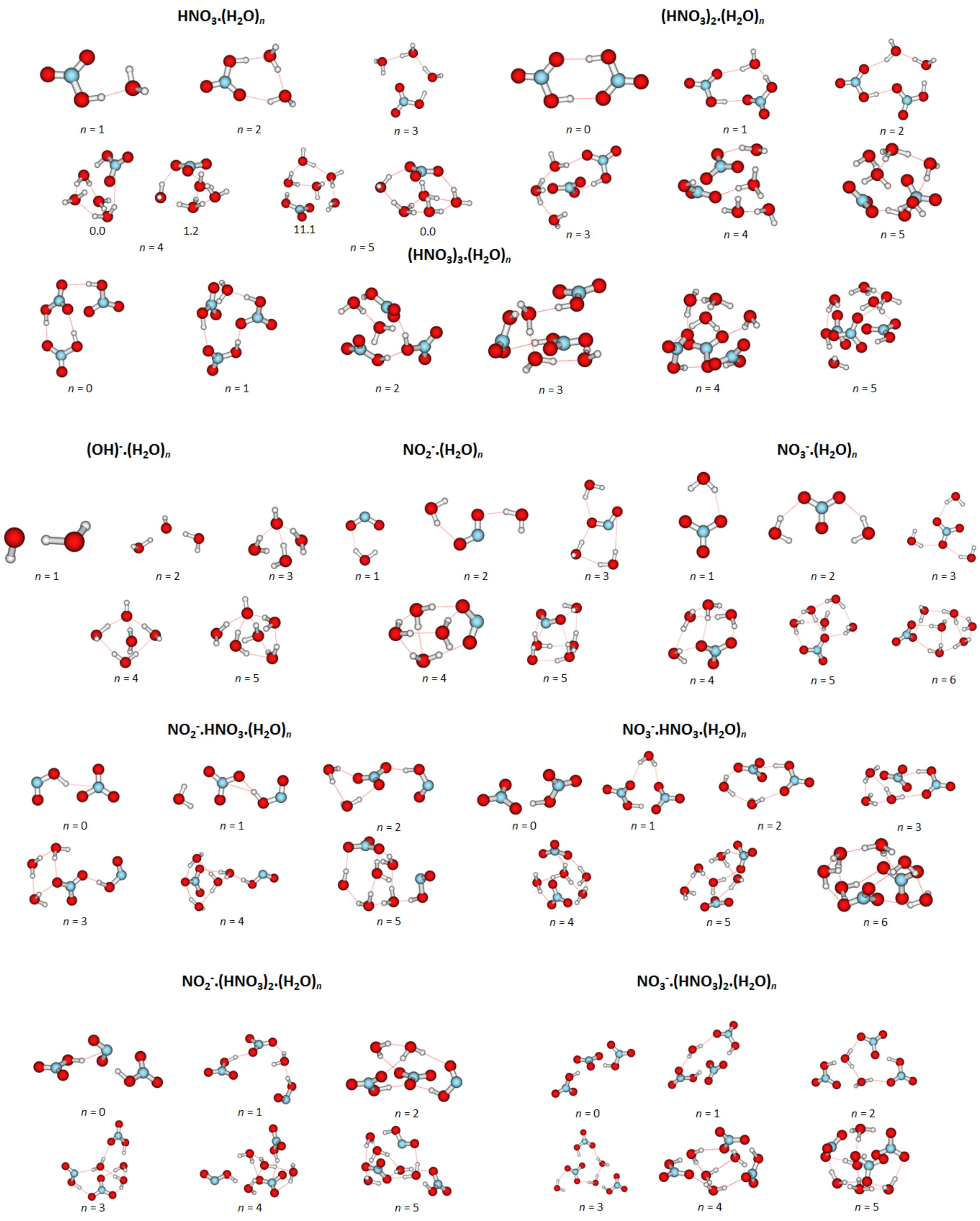

Figure S2. The most stable clusters optimized at the M06-2X/aug-cc-pVDZ level of theory. Both intact and ion-pair structure is included for $HNO_3(H_2O)_n$, n = 4,5, with relative energies given in kJ mol^{-1}.

Table S1 summarizes benchmark calculations of selected electron affinities and reaction enthalpies. Results of both B3LYP/aug-cc-pVDZ and M06-2X/aug-cc-pVDZ methods are in reasonable agreement with experiment and higher-level *ab initio* methods. With respect to the enthalpies of the HNO_3 + e^- reactions, the B3LYP/aug-cc-pVDZ energies are systematically shifted by about −0.2 eV with respect to the both experiment and CCSD values. Results of the M06-2X/aug-cc-pVDZ method lie much closer to the experimental values with the exception of the (NO_2 + OH^-) channel whose enthalpy is overestimated by about 0.3 eV because of the underestimation the OH electron affinity; there is no clear trend with respect to the CCSD results. Both functionals are able to reproduce enthalpy of HNO_3 reactions towards OH^- and NO_2^-.

Table S1. Electron affinities (EA) of OH and NO_2 and enthalpies of various reactions calculated at various levels of theory (all in eV). DZ and TZ stand for aug-cc-pVDZ and aug-cc-pVTZ, respectively. Enthalpies were calculated at 298.15 K within the harmonic approximation.

Property	B3LYP/DZ	M06-2X/DZ	M06-2X/TZ	MP2/DZ	CCSD/DZ	CCSD(T)/TZ*	exp.
EA(OH)	1.85	1.62	1.68	1.94	1.50	1.74	1.825[1]
EA(NO_2)	2.30	2.32	2.36	2.12	2.26	2.30	2.273[2]
Δ*H*(HNO_3 + e^- → NO_2^- + OH)	−0.34	−0.09	−0.12	0.07	−0.42	−0.19	−0.13[3]
Δ*H*(HNO_3 + e^- → OH^- + NO_2)	0.12	0.62	0.55	0.24	0.34	0.37	0.31[3]
Δ*H*(HNO_3 + e^- → NO_3^- + H)	0.28	0.37	0.35	0.37	0.45	0.49	0.45[3]
Δ*H*(HNO_3 + NO_2^- →NO_3^- + HONO)	−0.67	−0.74	−0.74	−0.60	−0.76	−0.69	−0.68[2,4]
Δ*H*(HNO_3 + OH^- → NO_3^- + H_2O)	−2.86	−3.05	−3.05	−2.72	−2.88	−2.83	−2.85[4,5]

* - calculated in the structure optimized at the CCSD/aug-cc-pVDZ level of theory, with respective thermal corrections also included at this level

Table S2 shows that there is a constant shift between B3LYP and M06-2X reaction energies with increasing hydration, with both methods predicting the same trends of reaction energies, with only mild differences. For example, the crossing point between NO_2^--forming and OH^--forming channel is predicted to be located between 0 and 1 water molecule for B3LYP/aug-cc-pVDZ and between 1 and 2 water molecules for M06-2X/aug-cc-pVDZ. The shift of the NO_3^--forming channel from endothermicity to exothermicity is predicted to take place for 1 water molecule (B3LYP) or between 1 and 2 water molecules (M06-2X).

Based on the calculations presented in Tables S1 and S2, we picked the M06-2X/aug-cc-pVDZ method for calculations performed in the main text, considering also its performance for investigating energetics of charged systems.[6] We note that, in the

present work, chemical trends with respect to hydration are of main concern, and a possible systematic shift of few tenths of eV does not influence our conclusions.

Table S2. Reaction energies (in eV) of various reactions calculated using M06-2X and B3LYP density functional with the aug-cc-pVDZ basis set.

reaction	functional	*n*			
		0	1	2	3
$HNO_3(H_2O)_n + e^- \rightarrow NO_2^-(H_2O)_n + OH$	M06-2X	−0.16	−0.47	−0.64	−0.79
	B3LYP	−0.41	−0.72	−0.91	−1.04
$HNO_3(H_2O)_n + e^- \rightarrow OH^-(H_2O)_n + NO_2$	M06-2X	0.54	−0.32	−0.78	−1.16
	B3LYP	0.04	−0.80	−1.30	−1.68
$HNO_3(H_2O)_n + e^- \rightarrow NO_3^-(H_2O)_n + H$	M06-2X	0.32	0.10	−0.07	−0.22
	B3LYP	0.22	0.00	−0.16	−0.26

Table S3 summarizes development of reaction energies for subsequent reactions of HNO_3, namely its reactions with OH^- to produce NO_3^- and H_2O and with NO_2^- to form HONO and NO_3^-. Both reaction energies become less exothermic with increasing hydration. However, they maintain their exothermic character even for five water molecules. In the molecular dynamics at the BLYP/6-31+g* level, both processes proceeded readily within 1 ps with the exception of $(HNO_3)_2NO_2^-$ where structure with NO_2^- is predicted to be the more stable one (see Figure S2).

Table S3. Reaction energies (in eV) of subsequent reactions of HNO_3 after first dissociation step takes place. Calculated at the M06-2X/aug-cc-pVDZ level of theory.

n	$HNO_3 + OH^-(H_2O)_n \rightarrow NO_3^-(H_2O)_{n+1}$	$HNO_3 + NO_2^-(H_2O)_n \rightarrow NO_3^-(H_2O)_n + HONO$
0	−3.69	−0.74
1	−2.96	−0.65
2	−2.60	−0.65
3	−2.34	−0.65
4	−2.11	−0.57
5	−1.91	−0.47

References

1. H. Hotop, T. A. Patterson and W. C. Lineberger, *J. Chem. Phys.*, 1974, 60, 1806.
2. K. M. Ervin, J. Ho and W. C. Lineberger, *J. Phys. Chem.*, 1988, 92, 5405.
3. N. S. Shuman, T. M. Miller and A. A. Viggiano, *J. Chem. Phys.*, 2012, 136, 124307.
4. J. A. Davidson, F. C. Fehsenfeld and C. J. Howard, *Int. J. Chem. Kinet.*, 1977, 9, 17.
5. J. R. Smith, J. B. Kim and W. C. Lineberger, *Phys. Rev. A*, 1997, **55**, 2036.
6. M. Walker, A. J. A. Harvey, A. Sen and C. E. H. Dessent, *J. Phys. Chem. A*, 2013, **117**, 12590.

Cartesian coordinates of molecules and ions used for calculations presented in Figure 2 (in Ångstrom) optimized at the M06-2X/aug-cc-pVDZ level of theory along with their electronic energies (zero-point energy corrected)

```
HNO3
-280.805724
N 0.138776 0.037639 -0.000279
O 0.119916 1.243468 0.000079
O 1.065182 -0.711515 0.000104
O -1.091292 -0.584310 0.000033
H -1.721880 0.155380 0.000232

HNO3.H2O
-357.208218
N 0.834817 0.070549 -0.002073
O 0.224409 1.123289 0.005944
O 2.018614 -0.086481 -0.022074
O 0.102051 -1.066357 0.013024
H -0.850531 -0.768275 0.021934
O -2.341628 -0.029342 0.077352
H -2.004829 0.874041 0.001182
H -3.015931 -0.128473 -0.602577

HNO3.(H2O)2
-433.609093
N -1.319356 -0.195189 -0.000029
O -2.483712 -0.466380 0.022761
O -0.386854 -0.983116 0.009661
O -1.023721 1.109103 -0.042130
H -0.007303 1.192041 -0.044091
O 1.517152 1.460003 -0.070423
H 2.013218 0.612564 -0.048172
H 1.876816 2.006168 0.635208
H 2.951071 -1.537813 -0.608795
O 2.480582 -1.058282 0.079927
H 1.574106 -1.397265 0.067687

HNO3.(H2O)3
-510.008505
O 0.252526 2.221684 -0.000488
O -1.879956 0.874716 0.085941
N -1.526843 -0.401626 -0.047105
O -2.403359 -1.213127 0.038340
O -0.340244 -0.625627 -0.245336
O 2.496360 0.775749 0.002131
H -1.002407 1.423023 0.048258
H 0.405357 2.874446 0.689328
H 1.060783 1.648357 -0.025153
H 2.379005 -0.192486 0.086598
H 3.069717 0.893682 -0.761015
H 1.090595 -1.940508 -0.031691
O 2.054774 -1.951722 0.060997
H 2.244043 -2.548506 0.790729

HNO3.(H2O)4
-586.408621
O -0.780741 -0.777275 -1.425262
O 1.541830 -1.081612 -0.649950
N 1.913129 -0.155092 0.211696
O 1.115686 0.757192 0.453227
O 2.999723 -0.267248 0.697506
O -2.643299 -1.495935 0.369458
O -1.673640 0.779572 1.521123
H 0.532446 -0.898070 -0.947370
H -1.457538 -1.180744 -0.832530
H -1.029577 0.158203 -1.508345
H -2.409365 -0.767607 0.981044
H -2.755542 -2.288915 0.899992
H -0.717715 0.672793 1.615229
H -1.750725 1.382886 0.767368
O -0.916897 1.997453 -1.003962
H -0.015091 1.882349 -0.664993
H -0.930095 2.827574 -1.489383

HNO3.(H2O)4, ion pair
-586.408167
N -0.233787 -0.124146 1.214288
O -1.325729 -0.667707 0.939276
O -0.168428 1.115442 1.333711
O 0.799205 -0.811760 1.318048
O 2.288389 0.869013 -0.285990
```

H 1.992436 0.481673 0.562783
H 3.171238 0.523574 -0.451556
H -1.270806 1.660166 0.048872
O -1.586522 1.631893 -0.884267
H -2.421396 1.151379 -0.832961
H -0.803838 -1.905512 -0.215458
O -0.321944 -2.178538 -1.030646
H 0.435045 -2.672012 -0.694010
H -0.508141 0.787920 -1.450376
H 0.082694 -0.795262 -1.499837
H 1.126303 0.475172 -1.217588
O 0.294152 0.186897 -1.733868

HNO3.(H2O)5
-662.809640
O 1.309802 -1.912702 0.092838
O -1.073091 -1.884817 -0.406382
N -1.580388 -0.770420 0.050849
O -2.636536 -0.403214 -0.406553
O -0.951099 -0.160160 0.915418
O 2.090573 -0.125038 -1.786667
O 1.790838 0.059657 1.911523
O 1.213784 1.966612 -0.150592
H -0.006456 -1.913262 -0.114668
H 1.710131 -1.445275 -0.671807
H 1.516285 -1.316719 0.851220
H 1.861808 0.703920 -1.327406
H 2.923824 0.031108 -2.238854
H 1.639126 2.824945 -0.060447
H 0.250352 2.157898 -0.234352
H 1.557187 0.767180 1.283562
H 1.029877 0.014273 2.501377
O -1.458038 2.411264 -0.150734
H -1.683856 1.663083 0.424628
H -2.025432 2.292970 -0.920013

HNO3.(H2O)5, ion pair
-662.813849
O 0.721819 0.733531 1.921055
O -0.632992 1.896215 0.117732
O -2.815416 0.607009 0.078335
O 0.821977 1.054074 -1.776547
O -0.820458 -1.110270 -1.223362
N -0.329133 -1.349585 -0.107973
O 0.869611 -1.655574 0.008585
O -1.042062 -1.259834 0.921337
H 0.318033 -0.151649 1.957919
H 1.602170 0.597068 1.531407
H -2.405770 -0.226776 0.403274
H -3.102128 0.389371 -0.816372
H 0.442212 0.167178 -1.922984
H 1.695146 0.896324 -1.378067
H -1.557205 1.438480 0.064807
H -0.075197 1.624812 -0.707595
H -0.120252 1.490854 0.914450
O 2.819895 0.214080 0.040626
H 2.363525 -0.644714 -0.039388
H 3.764403 0.032304 0.046268

(HNO3)2
-561.627741
O -1.222610 -1.012630 0.001128
N -2.009894 -0.073067 -0.000118
O -1.487412 1.167300 -0.002409
O -3.195381 -0.136442 0.000372
H -0.505189 1.050082 -0.001104
H 0.505185 -1.050116 0.000733
O 1.487429 -1.167305 -0.001043
N 2.009890 0.073063 0.000303
O 3.195407 0.136501 -0.001302
O 1.222571 1.012585 0.003140

(HNO3)2.H2O
-638.028583
O -0.866707 2.288495 0.152664
O -2.624019 0.491371 -0.336589
N -1.984885 -0.619604 0.025691
O -2.518234 -1.655016 -0.223473
O -0.904562 -0.466061 0.594387
H -1.977582 1.257077 -0.139547
H -0.054686 1.766503 0.258028
H -0.650899 3.009185 -0.447944
O 1.696488 0.908041 -0.017904
N 2.334062 -0.128580 -0.061034
O 3.504392 -0.253422 -0.245382
O 1.653533 -1.283874 0.115477
H 0.711809 -1.031746 0.263419

(HNO3)2.(H2O)2

-714.432474
O 0.467415 2.554127 0.151201
O -1.989290 1.051272 -0.057290
N -2.633709 0.016862 -0.018801
O -1.942338 -1.136166 0.058459
O -3.821068 -0.094988 -0.041315
O 0.637194 -0.368427 0.007218
N 1.454563 -1.296895 0.003823
O 2.727368 -0.967284 -0.050808
O 1.181513 -2.459509 0.044633
H 2.779318 0.086513 -0.071964
H -0.981368 -0.878785 0.046804
H 0.097347 3.294860 -0.338361
H -0.214947 1.866126 0.132481
H 3.430017 1.929157 0.544517
O 2.883887 1.512448 -0.129788
H 1.986197 1.930582 -0.067114

(HNO3)2.(H2O)3
-790.831672
O -0.531699 -2.176409 -0.740712
O -2.726500 -0.874680 -0.669975
O -1.650262 1.283390 -1.124719
N -0.602117 1.348890 -0.418889
O 0.270070 2.140011 -0.660198
O -0.499372 0.533523 0.559641
O 2.014482 0.365393 1.197953
N 2.593844 -0.285415 0.183825
O 3.779665 -0.403158 0.243481
O 1.864036 -0.703550 -0.707226
H -2.315503 0.101638 -0.883010
H 1.038488 0.470566 0.947469
H 0.211386 -1.563575 -0.590725
H -0.246019 -2.768647 -1.443655
H -2.954957 -0.825665 0.299611
H -1.912773 -1.477722 -0.738082
O -2.877736 -0.261372 1.850488
H -1.993735 0.135767 1.781171
H -2.910451 -0.741867 2.682802

(HNO3)2.(H2O)4
-867.236633
O 1.136221 -0.010473 2.346860
O 1.770364 0.441537 -2.289979
O -0.412599 1.501268 -1.153595
N 0.034217 1.715779 -0.014514
O 1.235815 1.892148 0.187605
O -0.766694 1.730813 0.967525
O 1.234880 -1.693045 -1.142967
O -2.680331 0.258213 0.347934
N -2.118777 -0.872687 -0.014494
O -0.929972 -1.040416 0.301783
O -2.789025 -1.663401 -0.606906
H 0.967254 0.972328 -2.069051
H -1.898756 0.916873 0.619532
H 1.314203 0.887204 2.022409
H 0.206454 -0.138486 2.110921
H 1.488801 -0.803756 -1.664342
H 2.470836 0.908023 -1.814875
H 1.831407 -1.751453 -0.277949
H 0.311515 -1.603403 -0.812633
O 2.576205 -1.643108 0.931403
H 2.068529 -1.043405 1.556209
H 2.832765 -2.433860 1.415538

(HNO3)2.(H2O)5
-943.645965
O 0.936442 0.726282 1.975916
O -1.651753 1.937637 1.197602
O -0.532565 2.141455 -1.075178
O -2.661745 -0.520682 0.040607
N -1.658145 -1.124279 0.434086
O -1.199694 -0.926858 1.568967
O -1.082400 -1.939899 -0.341673
O 1.334803 -1.943672 0.352751
N 1.883296 -0.961745 -0.348430
O 2.984870 -0.611659 -0.009813
O 1.237353 -0.476978 -1.264797
O 1.861078 2.158709 -0.228833
H -2.249398 1.171414 1.160546
H 0.335225 -2.015613 0.051649
H 0.401986 -0.080356 1.852161
H 1.567055 0.513763 2.672135
H -1.028107 2.101448 -0.176235
H -0.957566 1.677016 1.822343
H 0.469160 2.135458 -0.851301
H -0.811914 1.321617 -1.639828
H 2.489146 1.608776 -0.716201
H 1.711954 1.668928 0.603774

O -1.320726 0.117341 -2.373010
H -0.659619 -0.578546 -2.240989
H -2.089284 -0.215135 -1.877942

(HNO3)3
-842.442965
O -0.025978 1.005705 1.087927
N 0.375460 1.678897 0.155853
O 1.476137 2.142947 0.038074
O -0.468429 1.935350 -0.841727
O -2.698290 0.492765 -0.410967
N -2.795683 -0.650283 0.023727
O -3.715794 -1.386796 -0.109318
O -1.756956 -1.133743 0.732341
H -1.301732 1.435632 -0.631147
H -1.111980 -0.393668 0.810248
H 2.629867 0.621469 0.389684
O 3.117279 -0.226053 0.445456
N 2.348773 -1.129642 -0.227669
O 2.801493 -2.230058 -0.277368
O 1.306038 -0.719647 -0.693437

(HNO3)3.H2O
-918.852020
O -2.116629 -0.385156 -1.117939
N -2.715184 0.312314 -0.303750
O -2.001396 1.232450 0.359812
O -3.876679 0.275912 -0.054579
O 0.088121 -1.895649 -0.660010
N 0.324465 -1.745886 0.669646
O -0.556419 -1.270986 1.334608
O 1.413207 -2.117142 1.028222
O 0.595473 0.870530 -0.356363
N 1.384647 1.708941 0.096557
O 2.672922 1.462276 -0.071894
O 1.085142 2.713787 0.662451
H -1.058480 1.123948 0.067729
H 2.745247 0.530322 -0.514864
H -0.754940 -1.400043 -0.820146
H 2.809528 -1.509349 -0.411892
O 2.854504 -0.845606 -1.117825
H 2.035174 -0.995783 -1.609861

(HNO3)3.(H2O)2
-995.253212
O -1.608959 1.197629 2.072829
O -2.813223 -0.886491 0.839673
O -1.071418 -2.425131 0.002408
N 0.052710 -1.755917 0.024425
O 0.946753 -2.168040 -0.688843
O 0.131425 -0.771987 0.741563
O 2.095251 0.263674 -0.992894
N 2.628395 0.510934 0.257430
O 2.455606 1.636852 0.635667
O 3.185780 -0.400601 0.794130
H -1.816071 -1.782600 0.419205
H 1.941005 -0.715623 -1.008509
H -1.694828 2.001613 1.545571
H -0.668656 0.981331 2.039849
H -3.283992 -0.487052 0.096570
H -2.422351 -0.127941 1.340706
O -2.365303 1.535631 -0.789294
N -1.278313 1.113736 -1.088554
O -0.245142 1.853798 -0.625442
O -1.016301 0.126571 -1.729895
H 0.569597 1.353758 -0.865702

(HNO3)3.(H2O)3
-1071.653100
O -1.029608 2.777328 0.344430
O -0.396503 1.360892 -1.678593
O 2.093955 0.798517 -0.855759
N 2.315972 -0.536686 -0.994963
O 1.358400 -1.209529 -1.313400
O 3.433920 -0.890365 -0.772461
O -1.658012 -0.880832 -1.298052
N -2.564212 -0.545367 -0.372505
O -2.993437 0.579224 -0.370186
O -2.857743 -1.438990 0.394857
O -0.271691 -2.162073 1.261407
N 0.305313 -0.951566 1.310730
O -0.362632 0.011437 0.990637
O 1.447944 -0.936667 1.676408
H 1.159258 0.946038 -1.172073
H -1.220360 -0.011943 -1.578038
H -1.772441 2.361395 0.797871
H -0.248812 2.655716 0.928229
H -0.715097 1.957170 -0.914099
H -0.484685 1.868416 -2.492501

H -1.185926 -2.016604 0.916660
O 1.290329 2.282373 1.718146
H 2.100190 2.784192 1.584240
H 1.528997 1.360433 1.547386

(HNO3)3.(H2O)4
-1148.070819
O -0.558570 -1.198991 2.186988
O -2.165947 0.771118 1.821675
O -2.743455 -0.730106 -0.686067
N -1.810061 -0.175353 -1.238016
O -1.578973 1.010206 -1.208697
O -0.995937 -0.970117 -1.919608
O 1.285494 0.033556 -1.849297
N 1.912540 -0.643057 -0.945652
O 2.874324 -0.120276 -0.400875
O 1.508532 -1.764903 -0.652370
O 1.973244 -0.841365 2.251422
O 0.974200 2.461758 -0.811696
N 0.352002 2.112796 0.314308
O -0.326400 2.964349 0.828188
O 0.510047 0.980646 0.726552
H -2.725162 0.614971 1.046779
H -0.052837 -0.496759 -1.980065
H -0.738286 -1.876600 1.428057
H 0.439515 -0.982607 2.199450
H -1.724795 1.618859 1.655863
H -1.135497 -0.369625 2.043689
H 1.274423 1.600496 -1.228318
H 2.442335 -1.663766 2.429674
H 2.366433 -0.500514 1.427295
O -0.929728 -2.819572 0.288103
H -1.735612 -2.599755 -0.201857
H -0.206537 -2.615822 -0.329590

(HNO3)3.(H2O)5
-1224.478595
O 2.225417 0.343919 1.616599
O 2.361837 -2.092642 0.704858
O -0.124929 0.289520 2.470113
O 2.633567 2.019934 -0.232672
O -2.340386 -1.314889 1.031598
N -1.423332 -1.911004 0.475007
O -0.400673 -2.239223 1.090524
O -1.526632 -2.177285 -0.765349
O 0.703795 -1.880444 -1.765672
N 1.072000 -0.659757 -1.427194
O 0.327389 0.001251 -0.728155
O 2.157554 -0.301148 -1.834985
O -1.764124 1.772979 0.549024
N -0.793540 2.423941 -0.091949
O -0.870748 2.533658 -1.289431
O 0.093319 2.844197 0.623423
H 1.880682 2.631910 -0.207407
H -2.336191 1.263577 -0.172724
H 1.238416 0.383128 1.956646
H 2.365716 -0.575760 1.238632
H 2.511441 1.490580 -1.037007
H 2.394895 1.048660 0.878376
H -0.257906 -2.057313 -1.326389
H 1.449947 -2.430235 0.682015
H 2.765238 -2.346215 -0.133045
H -0.753444 0.880733 2.027436
H -0.476123 -0.605056 2.326733
O -3.104981 0.396098 -1.033618
H -3.095318 -0.453122 -0.553973
H -2.636506 0.229458 -1.860381

H2O
-76.387078
O 0.000000 0.000000 0.117469
H 0.000000 0.762125 -0.469876
H 0.000000 -0.762125 -0.469876

H
-0.497898
H 0.000000 0.000000 0.000000

NO2
-205.020427
N 0.000000 0.317838 0.000000
O 1.094434 -0.139651 0.000000
O -1.094434 -0.138457 0.000000

NO2-
-205.105813
N 0.000000 0.457100 0.000000
O 1.060962 -0.200470 0.000000
O -1.060962 -0.199492 0.000000

NO2-.H2O
-281.519498
N -1.281487 -0.010422 0.000274
O -0.641129 1.061298 -0.000334
O -0.606436 -1.063064 -0.000256
H 1.422247 0.761364 -0.000001
O 2.025605 0.000406 0.000351
H 1.323840 -0.677530 -0.000007

NO2-.(H2O)2
-357.926641
N 0.241998 0.786036 0.000041
O -0.865534 1.343744 -0.000087
O 0.231805 -0.470671 0.002628
H -1.825556 -1.343391 0.000800
O -2.666490 -0.866320 -0.001212
H -2.294566 0.036109 -0.000863
H 2.962720 0.460529 -0.002286
O 2.981083 -0.501744 -0.001024
H 2.016510 -0.695569 -0.000370

NO2-.(H2O)3
-434.331480
N 0.219005 -0.750546 0.021282
O 0.382167 0.496703 -0.105917
O 1.241764 -1.429640 0.091832
H 3.058557 -0.265345 0.055016
O 3.264683 0.677757 -0.019133
H 2.351035 0.999492 -0.093010
H -2.073371 1.890907 0.922795
O -2.004648 1.710690 -0.018977
H -1.111144 1.280839 -0.104270
H -2.797803 -0.144329 -0.130452
O -2.776570 -1.109906 -0.047412
H -1.819473 -1.272569 -0.002205

NO2-.(H2O)4
-510.738714
O -0.060488 2.024875 0.016761
O 1.584397 0.060634 -1.087141
N 2.253192 -0.229250 -0.062792
O 1.644166 -0.152331 1.018474
O -0.840817 -1.493813 -1.240682
O -2.519980 0.666573 -0.302043
H 0.446923 1.389802 -0.533765
H 0.225727 1.740956 0.894189
H -0.005313 -0.993942 -1.293536
H -0.902225 -1.696078 -0.292964
H -2.133119 -0.036891 -0.851788
H -1.784385 1.302718 -0.229990
O -1.037611 -0.993159 1.610922
H -1.634423 -0.354842 1.185765
H -0.142859 -0.649207 1.431309

NO2-.(H2O)5
-587.145344
O 2.629511 -0.207041 -0.520517
O 0.949002 2.085954 -0.577659
O -0.793704 0.027331 -1.716456
N -0.182442 -0.879737 -1.129810
O -0.861234 -1.612316 -0.374390
O -2.742777 0.294044 0.390109
O -0.342990 1.172678 1.742518
H 1.891136 -0.654733 -0.967785
H 2.291308 0.707856 -0.487360
H 0.409854 1.576011 -1.203380
H 0.513841 1.890899 0.277888
H -1.210768 0.903584 1.390798
H 0.144852 0.335969 1.857770
H -2.530657 0.762290 -0.428258
H -2.287727 -0.551600 0.200301
O 1.132991 -1.287527 1.693492
H 0.474725 -1.659393 1.081111
H 1.814136 -0.937701 1.090816

NO2-.HNO3
-485.975564
N -1.649246 -0.052240 -0.000192
O -1.635206 1.182179 -0.000366
O -2.695285 -0.700803 0.001143
O -0.536703 -0.685758 -0.001368
H 0.603344 0.203431 -0.000192
O 1.424107 0.889467 0.000454
N 2.589040 0.285841 0.000178
O 2.545350 -0.914915 0.000172

NO2-.HNO3.H2O
-562.380450

N -0.914775 0.455047 -0.006667
O -1.092616 -0.772296 -0.013847
O -1.861474 1.251150 -0.002372
O 0.267087 0.908076 -0.003155
H 1.309631 -0.209061 -0.003585
O -4.006888 -0.713422 0.013981
H -3.180914 -1.216792 0.000850
H -3.628190 0.178517 0.012113
N 3.233045 -0.587139 0.004142
O 1.981642 -1.004530 -0.003601
O 3.371198 0.602519 0.010032

NO2-.HNO3.(H2O)2
-638.781115
O -3.851656 -0.712745 -0.650508
H -3.065050 -1.005521 -0.167533
H -3.687173 0.239540 -0.713038
O -2.604678 1.841548 0.050153
H -2.681718 1.333602 0.867103
H -1.758920 1.492589 -0.289751
N -0.306755 -0.527633 0.304241
O -0.207252 0.426827 -0.485828
O -1.348888 -0.719202 0.951309
O 0.663683 -1.311909 0.448933
H 1.938105 -0.752895 -0.306749
O 2.794032 -0.573982 -0.842164
N 3.455533 0.459870 -0.336300
O 2.956422 0.945341 0.632403

NO2-.HNO3.(H2O)3
-715.183221
N 0.259732 -0.751461 -0.717899
O 0.207457 -1.779164 -0.041161
O 1.275483 -0.471679 -1.391383
O -0.716137 0.045338 -0.740643
O 3.067602 -1.443834 0.587099
O 3.051954 1.415274 1.420304
O 1.193209 2.296231 -0.722603
H 1.351296 1.470268 -1.209928
H 0.279092 2.167210 -0.441702
H 3.097604 0.468621 1.216389
H 2.437540 1.776982 0.762854
H 2.285083 -1.859004 0.970578
H 2.720954 -1.191372 -0.285079
N -3.759378 0.057972 0.680055
O -3.514528 1.084708 0.123210
O -2.776757 -0.835504 0.654531
H -1.980294 -0.469239 0.136960

NO2-.HNO3.(H2O)4
-791.593779
O -1.134058 -0.575730 -0.038772
O 1.255251 -0.847479 -1.518421
N 1.835049 0.194503 -1.149432
O 1.288008 1.295230 -1.271151
O 2.984389 0.110207 -0.637449
O -3.385509 0.591202 -0.211979
N -4.283821 -0.335854 0.108795
O -5.399359 0.080655 0.066568
O 2.343278 2.104196 1.260846
O 2.399458 -2.262838 0.742842
O 0.754604 -0.213739 1.901969
H -0.530404 -0.567671 -0.801162
H -2.473753 0.129191 -0.142565
H -0.541670 -0.383168 0.722934
H 1.715662 2.403379 0.588626
H 2.898432 1.519827 0.714921
H 1.710005 -2.472399 0.099646
H 2.870785 -1.554069 0.266078
H 1.281357 0.600166 1.808619
H 1.362495 -0.939436 1.671733

NO2-.HNO3.(H2O)5
-867.997413
O -0.281188 -2.751803 -0.134084
O -2.553622 -1.004571 -0.235674
N -1.997346 -0.192128 -1.009283
O -1.011460 -0.563496 -1.692523
O -2.401819 0.971960 -1.102008
O 4.364844 0.697677 -0.692211
N 3.311786 0.372998 -0.237853
O 3.418692 -0.446669 0.791494
O 1.081888 -0.971765 1.496327
O -0.444019 1.230732 1.510685
O 0.611552 1.727972 -1.030913
O -3.334190 1.011738 1.588530
H -3.395707 1.558619 0.793821
H -1.171821 -2.685003 0.235472
H -0.370742 -2.119198 -0.872699

H -1.412592 1.207775 1.593073
H -3.239301 0.135813 1.173966
H 0.470767 -0.195605 1.550189
H -0.239314 1.572627 0.618690
H 2.436033 -0.657493 1.081144
H 0.629891 -1.642901 0.943053
H 1.440047 1.250400 -0.880024
H 0.046245 1.094674 -1.503729

NO2-.(HNO3)2
-766.816100
O -3.015119 0.173955 1.095238
N -3.207812 -0.416888 0.049722
O -4.273858 -0.860379 -0.325191
O -2.182677 -0.605752 -0.761734
O -0.092636 0.376334 0.021634
N -0.096515 1.657700 -0.261750
O 0.929514 2.216894 0.014781
O 2.730672 -0.266985 -1.143067
N 3.017322 -0.603480 -0.014400
O 2.061510 -0.575515 0.910643
O 4.103224 -0.980443 0.368086
H -1.282970 -0.142023 -0.340166
H 1.206964 -0.144186 0.482044

NO2-.(HNO3)2.H2O
-843.222908
O 2.054599 0.461227 0.703405
N -0.728025 1.986675 -0.032357
O -0.778572 0.716893 0.120331
O 0.374049 2.543026 0.103022
O -1.745416 2.606641 -0.301872
H 1.392617 -0.236095 0.620315
H 1.539224 1.269829 0.487237
H -2.121053 0.228880 -0.061691
N -3.140365 -1.420598 -0.014695
O -4.218385 -1.953480 -0.157454
O -2.101644 -1.976742 0.283132
O -3.115002 -0.107246 -0.205938
H 3.425324 0.164096 0.054080
O 4.370652 0.065888 -0.338693
N 4.719234 -1.214280 -0.377150
O 3.885717 -1.967369 0.027752

NO2-.(HNO3)2.(H2O)2
-919.618495
N 0.803705 1.546013 -0.395121
O 0.441355 0.340423 -0.709709
O -0.072531 2.394074 -0.248016
O 1.996235 1.769295 -0.265529
H 1.836666 -0.549656 -0.939440
O -2.054846 0.193899 -1.175391
N -2.571245 -0.841866 -0.519334
O -3.763153 -0.978168 -0.608547
O -1.806538 -1.575223 0.081709
H -1.052067 0.241812 -0.936939
O 0.225739 -0.764210 2.074692
O -1.948036 1.122285 1.702928
H -1.185843 0.638880 2.057292
H -1.556658 1.686404 1.022475
H -0.305116 -1.067401 1.323217
H 1.084923 -0.566401 1.679669
O 2.642738 -1.074988 -1.241365
N 3.444894 -1.308670 -0.207484
O 3.018614 -0.946383 0.845141

NO2-.(HNO3)2.(H2O)3
-996.030120
O 0.288825 -2.328960 1.796593
O 1.353854 -1.610648 -0.583597
O 3.369910 -0.324954 -0.987962
N 3.508486 0.750992 -0.240354
O 4.516157 1.396731 -0.405540
O 2.624248 1.019255 0.560093
O -3.239432 1.664162 -0.630045
N -2.296783 2.054686 0.225325
O -2.245608 3.235110 0.342406
O -0.046312 0.427011 1.702756
O -3.162015 -0.917424 -0.426630
N -1.992708 -1.347073 -0.577798
O -1.717608 -2.511797 -0.278951
O -1.121379 -0.573031 -1.032407
H 2.430010 -0.835192 -0.741225
H -0.480692 -2.645397 1.298254
H 0.080053 -1.385863 1.953424
H 1.166336 -1.950088 0.323268
H 0.483918 -1.246747 -0.869917
H -3.179985 0.653244 -0.626361
H 0.763712 0.625699 1.211625

H -0.761449 0.770484 1.146988

NO2-.(HNO3)2.(H2O)4
-1072.431725
O -0.110763 -0.602513 2.058568
O -2.047109 -1.654586 0.554618
O 2.248684 -2.068298 1.484191
O -0.378264 1.546535 0.203247
O 2.484188 0.865376 0.908379
N 2.478317 1.745470 0.065696
O 2.670388 2.916104 0.255691
O 2.253699 1.390458 -1.201436
O 1.932058 -1.109869 -1.234059
N 0.708171 -1.440400 -1.233195
O 0.395080 -2.562038 -0.817638
O -0.143641 -0.642637 -1.640338
O -3.146915 0.543889 -0.026748
N -4.473968 0.407001 0.024147
O -5.041728 1.433666 -0.166835
H -0.272113 0.995925 -0.589055
H -1.347513 -1.358207 1.193767
H 0.736439 -1.080464 2.002048
H -1.333987 1.675811 0.257454
H 0.014929 0.221634 1.554522
H 2.095812 0.364899 -1.213783
H 1.835594 -2.552596 0.753230
H 2.628196 -1.300293 1.034323
H -2.743943 -0.388774 0.171762
H -1.526481 -2.011134 -0.181915

NO2-.(HNO3)2.(H2O)5
-1148.835680
O -2.081366 1.802684 2.020148
O -1.727458 3.102992 -0.452056
O 0.409772 1.365894 0.770158
O -3.380596 -0.397229 1.155642
O 4.880550 0.907388 0.518252
N 3.844773 0.341097 0.274560
O 3.012653 -0.009231 1.102082
O 3.601528 0.074797 -0.995431
O 1.542895 -1.217213 -1.292800
O 0.259651 -2.530506 0.823747
N -0.676282 -1.780356 0.809391
O -1.685660 -2.216142 1.525003
O -1.793194 -1.336110 -1.905681
N -1.681035 -0.142423 -1.675993
O -2.673486 0.565422 -1.385290
O -0.545105 0.418505 -1.720992
H 1.221366 0.888312 0.990974
H 2.670518 -0.471555 -1.062620
H -2.190211 2.424120 1.275871
H -2.979821 0.421395 1.542875
H 0.062672 0.915357 -0.019669
H -1.153850 1.535674 1.891789
H -3.386379 -0.209759 0.199586
H -2.417795 -1.489288 1.394479
H -0.780486 2.956532 -0.327213
H -2.016229 2.308557 -0.938068
H 0.742985 -0.714656 -1.582482
H 1.253579 -1.742933 -0.523485

NO3-
-280.296245
N -0.000043 0.000078 -0.000153
O -1.183569 0.400463 0.000045
O 0.244912 -1.225099 0.000045
O 0.938694 0.824568 0.000044

NO3-.H2O
-356.706697
O -2.503141 -0.008019 0.000056
O 0.150289 1.089354 -0.000238
N 0.770975 0.001126 0.000004
O 0.133076 -1.079636 0.000158
H -1.815812 -0.696026 0.000236
H -1.902765 0.753106 -0.000222
O 2.009994 -0.009818 0.000018

NO3-.(H2O)2
-433.113793
O -2.895794 -0.985624 -0.000912
O 1.078402 1.231356 -0.003525
N 0.000258 0.607280 -0.000312
O -1.078286 1.230774 0.003725
O 2.894998 -0.986744 0.001953
H -2.038536 -1.432039 -0.002049
H -2.569468 -0.069897 0.001625
H 2.034975 -1.428094 0.001847
H 2.573722 -0.069323 -0.000611

O 0.000367 -0.646213 -0.001070

NO3-.(H2O)3
-509.518597
O 2.572527 -2.126191 0.000094
O -0.230043 -1.237593 -0.000189
N -0.009060 -0.013298 -0.000511
O 1.168256 0.410442 0.000411
O -0.958228 0.797677 -0.001303
O 0.640288 3.261442 0.000781
H 1.654570 -2.426068 -0.000688
H 2.414296 -1.168864 0.000464
H -0.250168 2.886725 -0.002048
H 1.146174 2.433835 0.001684
H -2.408400 -1.669050 0.001307
O -3.167993 -1.072770 0.000583
H -2.691507 -0.227543 -0.000150

NO3-.(H2O)4
-585.922694
O 0.608602 2.113374 -0.383877
O -2.221556 1.562845 -0.061554
O -0.934334 -0.609368 1.150382
N -0.689819 -1.191430 0.058580
O -1.459152 -1.056368 -0.903865
O 0.335336 -1.890034 -0.035585
O 1.780341 0.351862 1.557610
H -0.359411 2.033907 -0.327018
H 0.935839 1.711567 0.440227
H 2.173769 0.014518 0.736302
H 0.963608 -0.170174 1.636462
H -2.297567 0.948887 -0.805173
H -1.935967 0.929538 0.623389
H 1.562394 -0.840598 -1.209933
O 2.165477 -0.094123 -1.351796
H 1.588362 0.686859 -1.274830

NO3-.(H2O)5
-662.325837
O -0.834225 -1.321298 -1.654923
O -2.999175 0.074849 -0.516995
O 0.278541 -2.571098 0.646798
O 0.668797 0.671254 -0.271792
N 1.927409 0.499976 -0.133944
O 2.339926 -0.630547 0.175504
O 2.688492 1.441529 -0.308883
O -1.196241 -0.259830 1.673908
H -0.581022 -1.984403 -0.986809
H -0.227806 -0.595704 -1.428280
H -0.461182 0.010059 1.092932
H -1.952178 -0.292917 1.058150
H -0.295657 -2.054547 1.233627
H 1.034598 -1.971555 0.499836
H -2.623542 0.970121 -0.521967
H -2.366003 -0.453468 -1.042257
O -1.440121 2.439037 0.257725
H -1.496857 1.962621 1.097185
H -0.570163 2.158808 -0.075549

NO3-.(H2O)6
-738.727092
O 0.513242 0.123178 1.407193
O 0.338197 -2.064643 -0.386014
O 3.380992 -0.061662 1.456160
O 2.097516 1.514145 -0.603775
O -0.598560 2.051150 -1.018080
O -1.938649 -0.366778 -0.919106
N -2.638207 -0.176422 0.117755
O -2.215377 0.629053 0.988368
O -3.702921 -0.757712 0.264004
O 2.832763 -1.115022 -1.248466
H -0.441534 0.324581 1.343024
H 0.929554 0.683430 0.729456
H -0.477852 -1.743019 -0.799488
H 0.427709 -1.449921 0.365735
H -0.992892 1.161756 -1.143932
H -1.000837 2.299407 -0.176705
H 2.531940 -0.280436 1.865811
H 3.155824 0.726089 0.936138
H 1.222761 1.823082 -0.909090
H 2.284904 0.689220 -1.092091
H 3.199067 -1.056542 -0.351737
H 1.971173 -1.556362 -1.113667

NO3.HNO3
-561.155057
O -1.125997 -0.867108 0.441237
N -1.894632 0.038370 -0.080020
O -3.047858 0.100598 0.324444

O -1.434629 0.773536 -0.945341
O 1.125113 -0.882279 -0.414756
N 1.895899 0.049161 0.082995
O 1.430767 0.810570 0.914921
O 3.046143 0.085065 -0.322781
H 0.042815 -0.775765 -0.002605

NO3.HNO3.H2O
-637.556255
O 0.048390 2.872411 -0.005823
O -1.309435 0.379738 0.889411
N -1.848559 -0.374541 0.089329
O -1.157738 -1.383200 -0.373363
O -2.992736 -0.263254 -0.303388
O 1.137816 -1.394708 0.371589
N 1.834987 -0.407017 -0.082173
O 2.988723 -0.296804 0.295801
O 1.302145 0.367511 -0.880627
H 0.667136 2.214315 -0.353742
H -0.638175 2.308303 0.372356
H -0.071271 -1.305270 -0.017508

NO3.HNO3.(H2O)2
-713.957257
O -1.119256 1.771133 0.921781
O -3.239102 1.276960 -1.025343
O -2.299916 -1.302547 0.195020
N -1.086191 -1.139312 0.246423
O -0.469229 -1.043988 1.310554
O -0.436126 -1.048591 -0.855702
O 1.968232 -0.901138 -0.560623
N 2.413809 0.271550 -0.180280
O 3.608348 0.366587 0.000377
O 1.614346 1.186728 -0.044386
H 0.859789 -0.890439 -0.629022
H -1.244453 1.012460 1.506214
H -0.263839 1.576702 0.509792
H -2.557301 1.612398 -0.421068
H -3.105897 0.322066 -0.962352

NO3.HNO3.(H2O)3
-790.361863
O 3.301426 0.690073 -1.158944
O 0.927774 1.904593 -0.210089
O 1.830762 -1.592579 -0.161247
N 0.647356 -1.243439 -0.204135
O 0.099491 -0.874134 0.901667
O -0.010745 -1.225891 -1.233131
O 2.636994 0.621386 1.714517
O -2.339505 -0.716303 0.697962
N -2.773731 0.323081 0.010350
O -1.965246 1.161460 -0.348841
O -3.964844 0.364284 -0.199782
H -1.259672 -0.709227 0.718239
H 2.984462 -0.223841 -1.191389
H 2.471778 1.197986 -1.093861
H 0.044735 1.515597 -0.280506
H 1.275625 1.587313 0.640434
H 2.101662 -0.184111 1.665430
H 3.137172 0.595687 0.881254

NO3.HNO3.(H2O)4
-866.769205
O -0.103180 -2.623506 -0.106188
O 2.362822 0.816209 1.203439
N 1.632943 -0.183478 1.067712
O 2.136501 -1.256104 0.634431
O 0.421605 -0.124478 1.314428
O -2.409055 -0.951536 0.232524
N -2.364719 0.282763 0.199284
O -2.918302 0.996182 1.012547
O -1.712013 0.839619 -0.768595
O -0.229088 -0.785819 -1.864029
O 1.949653 0.610422 -1.762094
O 0.602850 2.474464 -0.269340
H -0.928042 -0.106835 -1.397248
H 0.717836 -2.277562 0.312707
H -0.813549 -2.199297 0.403833
H 0.666291 -0.310509 -1.857763
H -0.167727 -1.592437 -1.227689
H 1.563441 1.390243 -1.295297
H 2.545537 0.216498 -1.107672
H 1.013601 2.133224 0.543170
H -0.289303 2.088062 -0.259997

NO3.HNO3.(H2O)5
-943.174569
O 0.200959 -0.144231 -2.271859
O 0.905007 1.863931 -0.851434

O -0.656291 1.783938 1.109064
O -0.056361 -0.688025 2.072404
O -3.366683 1.642359 0.401017
O -2.378646 -0.415232 -1.254817
N -2.053968 -1.148412 -0.280124
O -2.680413 -1.061328 0.796068
O -1.108457 -1.933714 -0.391728
O 4.023870 -0.818177 0.560360
N 3.038749 -0.226432 0.161935
O 1.941033 -0.802470 -0.012745
O 3.114070 1.021527 -0.091786
H 1.843439 1.516952 -0.505210
H -0.747344 -0.327392 -2.112579
H 0.646318 -0.787428 -1.696638
H 0.245668 1.865094 -0.045559
H 0.584164 1.123079 -1.476267
H -1.611016 1.816116 0.894698
H -0.492355 0.912560 1.543037
H -3.171647 1.222005 -0.453887
H -3.496000 0.839806 0.929851
H 0.628512 -0.934476 1.427744
H -0.827924 -1.211031 1.805786

NO3.HNO3.(H2O)6
-1019.578698
N -2.514816 -0.837865 -0.034853
O -2.896555 -1.052677 -1.172126
O -2.869542 0.159087 0.617486
O -1.711851 -1.660539 0.531095
O -0.286922 -0.279996 2.084382
O 0.627506 -2.481265 -0.876531
O 1.842543 -1.401441 1.317308
O 4.048918 0.121059 0.377602
N 1.292755 0.671033 -1.040513
O 1.724884 1.634885 -0.362615
O 0.074340 0.458212 -1.122071
O 2.097692 -0.082720 -1.624075
O -0.344557 2.145413 1.395548
O -2.069674 2.409896 -0.794892
H -1.359437 1.887156 -1.199356
H -2.685328 1.716746 -0.501794
H -0.291073 -2.286252 -0.618236
H -0.956308 -0.831686 1.519283
H -0.373800 0.720590 1.812840
H 0.858098 -1.759359 -1.480791
H 0.636736 -0.640685 1.808440
H 1.523530 -1.868694 0.508556
H 2.658733 -0.927929 1.068252
H 3.670351 -0.027800 -0.507695
H 3.660576 0.982415 0.580151
H -1.097215 2.318452 0.792204
H 0.415293 2.125548 0.776819

NO3.(HNO3)2
-841.991793
O -0.002576 0.136741 0.072150
N -0.002854 1.441171 -0.015346
O 0.976153 1.974423 -0.510301
O -0.982564 2.034823 0.404781
O 2.943034 -0.095267 1.114236
N 3.171056 -0.671009 0.073292
O 2.153156 -0.883721 -0.761054
O 4.242874 -1.093192 -0.297628
O -2.848315 -0.215906 -1.146074
N -3.163157 -0.675420 -0.070477
O -4.260534 -1.070955 0.251828
O -2.221197 -0.774483 0.867953
H 1.318503 -0.411271 -0.369206
H -1.354066 -0.351636 0.489794

NO3.(HNO3)2.H2O
-918.392133
O -0.046555 -0.883419 -2.205004
O -4.374456 -0.770689 0.668748
N -3.218067 -0.583572 0.374026
O -2.710009 -0.810649 -0.706430
O -2.447427 -0.092413 1.338304
O -0.110690 0.350817 0.566419
N -0.032221 1.398710 -0.147033
O -1.028851 2.000127 -0.488935
O 1.115838 1.785705 -0.479340
O 2.973593 0.619065 0.727655
N 3.199536 -0.630817 0.334965
O 4.077035 -1.214421 0.925206
O 2.528644 -1.076236 -0.574186
H -1.497975 0.091833 0.932993
H 2.163908 0.993940 0.184943
H 0.697360 -0.830512 -1.592031
H -0.825009 -0.808607 -1.639103

NO3.(HNO3)2.(H2O)2
-994.802175
O 0.410149 1.425838 1.383281
O 1.669459 -0.938898 1.235448
O 3.719060 -0.492825 0.022527
N 3.759039 0.711942 -0.497072
O 2.810831 1.461933 -0.301170
O 4.744877 0.998636 -1.136710
O -3.331854 0.068328 -0.458382
N -3.307645 1.358003 -0.188066
O -4.309590 1.981744 -0.443685
O -2.288407 1.830719 0.296028
O -0.039015 -2.745035 0.095504
N -1.094007 -2.140850 -0.144426
O -1.138583 -0.890299 0.142480
O -2.069981 -2.684470 -0.632906
H 2.744640 -0.649275 0.553544
H -0.468146 1.346369 0.980956
H 0.987342 1.752150 0.680308
H 1.076728 -1.597192 0.805166
H 1.117472 -0.126995 1.361894
H -2.375325 -0.354090 -0.194234

NO3.(HNO3)2.(H2O)3
-1071.200550
O -1.855577 4.845241 -0.344643
O 1.683867 0.741944 1.269059
O 3.746868 0.699863 -0.024911
N 4.021969 -0.492188 -0.510884
O 5.036019 -0.591307 -1.160008
O 3.246269 -1.407969 -0.273260
O -1.127221 0.297768 0.237888
N -1.317843 1.540356 0.043419
O -2.407698 1.933250 -0.358437
O -0.376419 2.316725 0.273056
O 0.812747 -1.803150 1.324277
O -3.184343 -1.011046 -0.326957
N -2.880964 -2.298266 -0.253505
O -3.765196 -3.069835 -0.528425
O -1.747374 -2.605321 0.081309
H 2.797930 0.662757 0.531008
H -1.013512 4.462291 -0.070763
H -2.377238 4.040911 -0.470683
H 1.270687 -0.150947 1.363842
H 0.982201 1.316309 0.896941
H -2.318662 -0.459095 -0.090253
H 1.455025 -2.030354 0.639286
H -0.054110 -1.860483 0.895823

NO3.(HNO3)2.(H2O)4
-1147.605157
O -0.188246 -2.456646 -1.297602
O -1.609792 -0.279293 -1.722605
O 2.342354 -1.346975 -1.896133
O -0.462725 -1.655531 1.338450
O 0.760823 0.855065 -0.798354
N 0.916623 2.057062 -0.501032
O 0.063228 2.893105 -0.693793
O 2.032771 2.406934 0.028857
O 1.906537 0.005014 1.901395
N 2.747601 -0.424352 1.127564
O 3.051194 -1.596679 1.011279
O 3.378122 0.427634 0.356704
O -2.732115 0.231261 1.102510
N -3.816886 0.103829 0.550251
O -4.890684 0.224071 1.089180
O -3.829969 -0.180939 -0.735639
H 0.309402 -1.165064 1.656496
H -1.129007 -1.152316 -1.636317
H 0.716877 -2.213662 -1.568311
H -1.158750 -0.988417 1.246696
H -0.193785 -2.335024 -0.326394
H 2.731351 1.362273 0.252906
H 2.032873 -0.459999 -1.655540
H 2.945308 -1.572117 -1.174700
H -2.797728 -0.218201 -1.109861
H -0.959892 0.370585 -1.396462

NO3.(HNO3)2.(H2O)5
-1224.018214
O -0.338729 1.477437 2.108732
O -1.459283 -0.802967 2.482067
O -1.248670 2.451459 -0.027492
O 2.164815 1.300837 1.731724
O 2.664017 -1.468775 1.176945
N 1.657174 -1.626968 0.486624
O 1.663679 -2.450003 -0.466499
O 0.619312 -0.970810 0.710432

O 2.279427 0.379875 -1.305640
N 1.372893 1.197928 -1.392659
O 1.437068 2.302328 -0.801217
O 0.335752 0.949643 -2.068410
N -2.680148 -0.440733 -0.393157
O -3.316343 -0.037827 0.592141
O -2.214313 -1.612968 -0.392734
O -2.476761 0.294712 -1.369869
O -0.168805 -1.556430 -1.967752
H -1.798777 1.830193 -0.543079
H -1.015743 -1.593124 -1.400439
H -0.771634 0.569012 2.293243
H 0.682391 1.356070 1.965120
H -0.431476 2.547138 -0.553128
H -0.753732 1.866022 1.235780
H 2.407779 0.359752 1.627998
H 2.250684 1.674377 0.835034
H -2.297575 -0.731317 1.986207
H -0.887244 -1.316539 1.887489
H 0.581695 -1.963895 -1.379754
H 0.054885 -0.561367 -2.079568

OH
-75.705909
O 0.000000 0.000000 0.108376
H 0.000000 0.000000 -0.867005

OH-
-75.765361
O 0.000000 0.000000 0.107377
H 0.000000 0.000000 -0.859016

OH-.H2O
-152.199421
O 1.223559 0.093215 -0.064725
H 1.526104 -0.594697 0.536443
H -0.077818 -0.022669 -0.071792
O -1.220485 -0.096912 -0.055669
H -1.472879 0.646940 0.498502

OH-.(H2O)2
-228.617245
O -0.003550 0.741993 0.066453
H -0.001797 1.459404 -0.573587
H -1.301401 0.033947 -0.063685
O -2.237148 -0.436641 -0.095237
H -2.595448 -0.239594 0.773789
H 1.363012 0.134631 0.070732
O 2.296675 -0.331142 0.046304
H 2.087816 -1.182061 -0.347409

OH-.(H2O)3
-305.030801
O -0.009180 0.000267 1.424755
H -0.023860 -0.002531 2.382325
H 0.957484 0.851633 0.368091
O 1.392526 1.104804 -0.497137
H 1.428657 0.235261 -0.916983
H 0.253408 -1.251767 0.364958
O 0.268068 -1.752884 -0.502601
H -0.502735 -1.355947 -0.928729
O -1.649292 0.647966 -0.511935
H -1.212937 0.403398 0.356591
H -0.916997 1.118730 -0.930905

OH-.(H2O)4
-381.442720
O -0.002136 -1.468778 0.479116
O 0.000021 -0.131342 -1.885454
O -2.121308 -0.058121 0.548814
O 2.120784 -0.062204 0.548914
H -0.002008 -2.410327 0.660503
H -1.327815 -0.703084 0.613533
H -2.252762 -0.007897 -0.404610
H 1.325689 -0.704901 0.614155
H 2.250989 -0.011859 -0.404694
H -0.000667 -0.775895 -1.135976
H 0.000652 0.716048 -1.415160
O 0.002648 1.877687 0.348519
H -0.764206 1.319700 0.576621
H 0.770056 1.320279 0.576350

OH-.(H2O)5
-457.851218
O 0.287598 -0.581891 -1.307571
O -0.211735 -1.396565 1.285221
O -1.289129 1.333436 1.150403
O 0.969834 1.843318 -0.498972

O 2.505710 -0.583831 0.303349
H 0.453005 -0.983763 -2.163698
H -0.016516 -1.312023 0.326478
H -0.544529 -0.507531 1.496875
H -1.799739 0.885673 0.451756
H -0.503978 1.658110 0.668131
H 0.668161 1.020467 -0.976075
H 1.722340 1.490420 -0.004757
H 2.004975 -0.896385 1.068602
H 1.812031 -0.634286 -0.399450
H -1.316234 -0.507319 -1.119777
O -2.285220 -0.493933 -0.831937
H -2.295975 -1.177635 -0.152022
